# BACE1-Dependent Neuregulin-1 Signaling: An Implication for Schizophrenia

**DOI:** 10.3389/fnmol.2017.00302

**Published:** 2017-09-25

**Authors:** Zhengrong Zhang, Jing Huang, Yong Shen, Rena Li

**Affiliations:** ^1^National Clinical Research Center for Mental Disorders, Beijing Key Laboratory of Mental Disorders, Beijing Anding Hospital, Capital Medical University Beijing, China; ^2^Neurodegenerative Disorder Research Center, School of Life Sciences, University of Science and Technology of China Hefei, China; ^3^Center for Therapeutic Strategies for Brain Disorders, Roskamp Institute, Sarasota FL, United States; ^4^Center for Hormone Advanced Science and Education, Roskamp Institute, Sarasota FL, United States; ^5^Beijing Institute for Brain Disorders, Capital Medical University Beijing, China

**Keywords:** schizophrenia, β-secretase (BACE1), neuregulin-1 (NRG1), erb-b2 receptor tyrosine kinase 4 (ErbB4), signaling pathway

## Abstract

Schizophrenia is a chronic psychiatric disorder with a lifetime prevalence of about 1% in the general population. Recent studies have shown that Neuregulin-1 (Nrg1) is a candidate gene for schizophrenia. At least 15 alternative splicing of NRG1 isoforms all contain an extracellular epidermal growth factor (EGF)-like domain, which is sufficient for Nrg1 biological activity including the formation of myelin sheaths and the regulation of synaptic plasticity. It is known that Nrg1 can be cleaved by β-secretase (BACE1) and the resulting N-terminal fragment (Nrg1-ntf) binds to receptor tyrosine kinase ErbB4, which activates Nrg1/ErbB4 signaling. While changes in Nrg1 expression levels in schizophrenia still remain controversial, understanding the BACE1-cleaved Nrg1-ntf and Nrg1/ErbB4 signaling in schizophrenia neuropathogenesis is essential and important. In this review paper, we included three major parts: (1) Nrg1 structure and cleavage pattern by BACE1; (2) BACE1-dependent Nrg1 cleavage associated with schizophrenia in human studies; and (3) Animal studies of Nrg1 and BACE1 mutations with behavioral observations. Our review will provide a better understanding of Nrg1 in schizophrenia and a potential strategy for using BACE1 cleavage of Nrg1 as a unique biomarker for diagnosis, as well as a new therapeutic target, of schizophrenia.

## Introduction

Schizophrenia is a hereditary, disabling mental disorder that affects ∼1% of the general population. The etiology of schizophrenia is complicated and is influenced by more than genetics alone. Other factors such as neurotransmitter imbalance, abnormal neuronal development, infection, and neuronal inflammation are also possible mechanisms (Schultz et al., 2007). Schizophrenia is characterized by several major clinical symptoms such as positive symptoms (hallucinations and delusions), negative symptoms (emotional blunting, and social withdrawal), and cognitive impairments (attention, performance, and working memory). *NRG1* was one of the 108 schizophrenia-associated genes identified in 2014 (Schizophrenia Working Group of the Psychiatric Genomics Consortium, 2014), and it attracted much attention due to its role in regulation of neuronal migration and myelination. *NRG1* is widely distributed in the frontal cortex, midbrain, and cerebellum (Rieff et al., 1999; Liu et al., 2001; Stefansson et al., 2003), and significantly associated with endophenotypes of schizophrenia via regulating myelination (Chen et al., 2006), neuronal migration (Ghashghaei et al., 2006), and function of neurotransmitter receptors (Liu et al., 2001; Hahn et al., 2006). Nrg1 can be cleaved by the proteolytic enzyme, BACE1. The BACE1-cleaved Nrg1-ntf plays roles in brain function via activation of ErbB receptor signaling pathways (Luo et al., 2011). Since most studies have compared the total Nrg1 levels between schizophrenia and healthy controls, it is critical to know whether the specific activity of BACE1 in cleavage of Nrg1 plays an important role in schizophrenia. In this review, we provide a summary and perspective on information of BACE1 involvement in Nrg1 regulation in schizophrenia according to recent clinical and preclinical discoveries, presented in three sections: (1) Nrg1 structure and cleavage pattern by BACE1; (2) BACE1-dependent Nrg1 cleavage associated with schizophrenia in human studies; (3) Animal studies of Nrg1 and BACE1 mutations with behavioral observations.

### Neuregulin-1 Structure and Cleavage Pattern by BACE1

**FIGURE 1:**
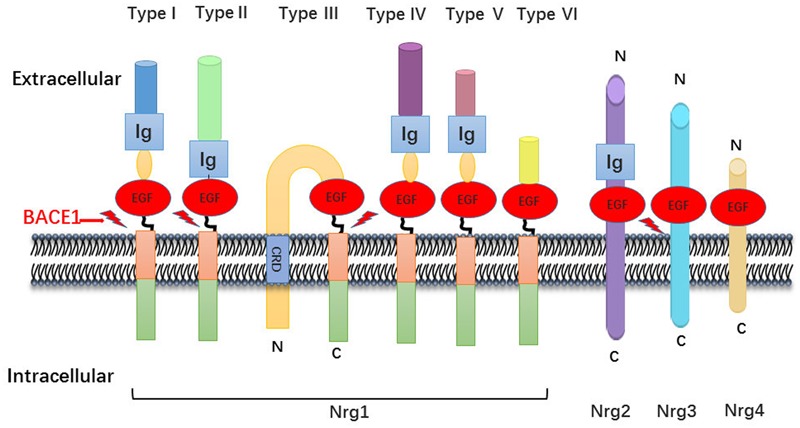
Neuregulin-1 isoforms and membrane location. Nrg1 has six different forms, which distinguish mainly from their N-terminal peptide. All six isoforms own an EGF-like domain and contain the Ig domain excluding type III Nrg1. There is a cysteine rich domain (CRD) embedded in the lipid bilayer in the type III Nrg1, which leaves N-terminal side tethered on the membrane. As described, the other family member Nrg2, Nrg3, Nrg4 has the similar domain anchor once membrane.

The neuregulin family includes four proteins (Nrg1, Nrg2, Nrg3, and Nrg4), encoded by their respective genes, which are widely expressed in various tissues including brain, heart, and breast. In general, Nrg1 can be divided into three major isoforms from alternative splicing. Type I Nrg1 has alternative names such as acetylcholine receptor inducing activity, differentiation factor, or neuregulin. Type II Nrg1 is also called glial growth factor, while type III Nrg1 is also known as sensory and motor neuron-derived factor. There are common structures between Nrg1 isoforms, such as Ig domains, EGF domains, a transmembrane region and unequal length of intracellular domain (Falls, 2003). Due to alternative splicing effect, Nrg1 is also divided into type alpha and beta based on the difference between the 5th and 6th cysteine amino acid in the EGF-like domain, whereas the beta variant has higher affinity for its downstream ErbB receptors (Wen et al., 1994; Burgess et al., 1995) (**Figure 1**).

Nrg1-induced cellular responses are mostly mediated by binding to tyrosine kinase receptors in the ErbB family. The ErbB family includes ErbB1, ErbB2, ErbB3, and ErbB4 receptors. Nrg1-mediated ErbB2 receptor activation requires the participation of ErbB3 or ErbB4 to form heterodimers (Bublil and Yarden, 2007). ErbB3 on its own lacks tyrosine kinase activity, so the activation of ErbB3 is dependent on heterodimer formation with other ErbB receptors (Falls, 2003). Nrg1 performs most of its functions via binding to both ErbB3 and ErbB4, while Nrg3 can only bind to ErbB4 (Zhang et al., 1997).

**FIGURE 2:**
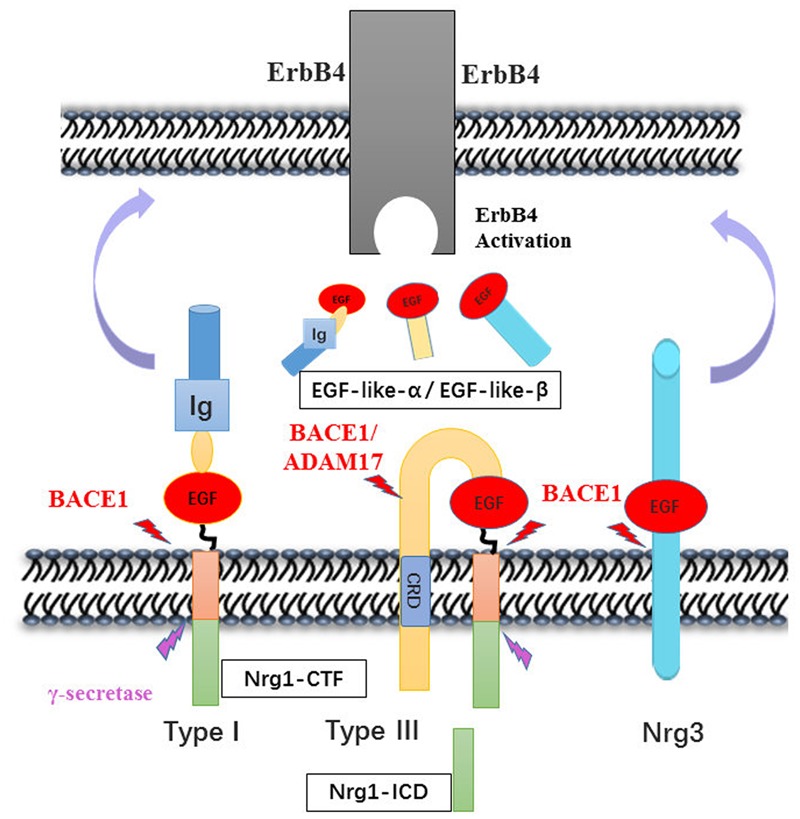
Neuregulins proteolytic cleavage pattern by BACE1. The enzymatic mapping shows that the cleavage of Nrg1 is mediated by BACE1 at between Glu-Phe and Met-Glu. Then, type I Nrg1 releases its N-terminal fragment to the extracellular space, in which the Nrg1-ntf binds to ErbB4 receptor on nearby cells. Whereas type III Nrg1 was cleave by BACE1 remaining tethered on the lipid bilayer via its hydrophobic Cys-rich domain. The second may cut may occur by either BACE1 or ADAM10/17 cleavage and release the EGF-domain that signal through ErbB4 in adjacent glial cells. Nrg3 was also cleaved by BACE1, producing the EGF-domain to activity its downstream pathway. The Nrg1-CTF was cleaved by γ-secretase in the cytosolic space, producing C-terminal peptide Nrg1-ICD that may regulate neuron development.

Both human and animal studies have shown that BACE1-cleaved Nrg1-ntf plays roles in brain function via activation of ErbB receptor signaling pathways. BACE1 cleaves type I and III Nrg1 at its position between the region of EF and ME residues and releases soluble fragments of Nrg1. BACE1, together with ADAM17 or ADAM10 which is also called TACE was involved in successive release of the EGF-like domain of NRG1 type III two membrane-bound structures, which has been generated by an initial BACE1 dependent proteolytic cleavage (Horiuchi et al., 2005). NRG3, another substrate of BACE, was considered as a compensation for loss of NRG1 and cleaved to produce EGF-domain through juxtacrine interactions with ErbB4 receptor like NRG1-CRD on axon of neuron (Vullhorst et al., 2017). These fragments bind to the ErbB4 receptor at its EGF-like domain, thereby activating ErbB receptors involved in Nrg1/ErbB signaling pathways that ultimately increase ERK and AKT phosphorylation, which are necessary for cell survival, synaptic development, glutamatergic transmission (Krivosheya et al., 2008; Mei and Xiong, 2008), and remyelination (Hu et al., 2006; Luo et al., 2011). The remain fragment of NRG1 cleavage is called NRG1-CTF, which can be further processed by γ-secretase to release the NRG1-ICD that participated to enhance synaptic plasticity for the development of cortical neurons (Bao et al., 2004; Chen et al., 2010). In addition, it is speculated that the expression of NRG1-CTF might be regulated by antipsychotic drugs, as the same effect on NRG1 precursor (Hashimoto et al., 2004; Barakat et al., 2010) (**Figure 2**).

Nrg1/ErbB signaling pathways are important in the regulation of the central nervous system, particularly in regulation of neuronal migration, myelination and glutamatergic networks. For example, during cortical development, neuronal Nrg1 reacts with ErbB4 in glial cells to promote cerebral cortical neurons and cerebellar granule cell migration (Schmid et al., 2003). By blocking ErbB in glial cells, both radial glia formation and neuronal migration, were impaired (Rio et al., 1997). Another important function of Nrg1/ErbB signaling is helping myelin formation. The dysfunctions of myelination have been reported in the PNS of schizophrenic patients (Chavarria-Siles et al., 2016; Stedehouder and Kushner, 2017). The activation of Nrg1/ErbB has involved both formation of myelin and development myelination via axonal signaling in Schwann cells, such as Nrg1-type III which is interacting with ErbB2 and ErbB3 (Boerboom et al., 2017; Miyamoto et al., 2017). In addition, Nrg1 is required for post-injury remyelination in later adulthood (Stassart et al., 2013). Some reported indicated that Nrg1 can regulate Schwann cell development to promote myelination (Michailov et al., 2004; Nave and Salzer, 2006), as well as affect oligodendrocyte proliferation or differentiation (Fernandez et al., 2000; Flores et al., 2000). For example, the development of oligodendrocytes was paused at the pre-oligodendroblast stage in ErbB2-null mice, which indicated that Nrg1/ErbB was an essential integrant in the final step of oligodendrocyte differentiation (Park et al., 2001). Lastly, Nrg1/ErbB signaling can directly affect glutamatergic systems by regulating the expression and function of N-methyl-D-aspartate (NMDA) receptors with brain region specificity. For example, in the prefrontal cortex, Nrg1 may promote NMDA receptor type 1 subunit endocytosis and inhibit NMDA receptor-mediated activity in prefrontal cortical pyramidal neurons (Yarden and Sliwkowski, 2001). Nrg1 type β significantly increased levels of NMDA receptor type 2C subunit in the cerebellum (Harrison and Law, 2006), while also reversing the long-term potentiation in the hippocampal CA1 region through regulation of AMPA endocytosis (Kwon et al., 2005). Importantly, Nrg1 and Nrg1/ErbB signaling regulate several processes of neurodevelopment that play extremely critical roles in schizophrenia neuropathology.

*NRG3*, a paralog of *NRG1*, was also reported its risk variants associated with clinical symptoms and cognitive function (Kao et al., 2010; Diez et al., 2014). Moreover, genetic modified mice of Nrg3 also exhibit behaviors consistent with psychotic disorders (Hayes et al., 2016). Nrg3 is a critical mediator in the assembly of cortical inhibitory circuits and balance of ex-inhibition, which is hypothesized as pathophysiology schizophrenia (Bartolini et al., 2017). As the closest NRG1 homolog, NRG2 is involved in increasing susceptibility to schizophrenia from human study through interaction with other NRG and ERBB (Benzel et al., 2007). Nrg2 has also been involved in the modulation of schizophrenia-liked behaviors in animal studies (Yan et al., 2017). In addition, *in vitro* studies showed that Nrg2 plays roles in dopamine system regulation, bidirectional mediation of GABAergic synaptogenesis and maturation of glutamatergic synapse in network integration of newborn neurons (Oh et al., 2015; Yan et al., 2017). Comparing other members of NRG family, there are no direct evidence of Nrg4 linked to schizophrenia. The function of Nrg4 has been mainly reported in modulating of energy metabolism and the development of obesity-associated disorders (Wang et al., 2014; Jiang et al., 2016).

### Human Studies: BACE1-Dependent NRG1 Cleavage in Schizophrenia

As a member of the neuregulin family, NRG1 is a key molecule involved in normal brain development. Limited human studies on NRG1 in schizophrenia have shown that the structure and biological process of NRG1 is associated with disease susceptibility as well the clinical phenotypes. In this section, we will focus on the function of BACE1-dependent NRG1 cleavage in schizophrenia clinical studies.

#### NRG1 Proteolysis in Schizophrenia

BACE1 has at least 15 well-known physiological substrates, as numerous novel substrates were identified by means of different screens (Kuhn et al., 2012; Zhou et al., 2012). Activity of BACE1 has cell-, tissue-, and substrate-specificity. Early studies of postmortem schizophrenia brains showed no differences of BACE1 protein levels in Brodmann’s area (BA) 6 compared to control subjects (Dean et al., 2008). It is suggested that only measuring BACE1 protein levels in the brain might not be sufficient to show BACE1-specific activity in cleaving NRG1 in schizophrenia. Later, the same research group reported a positive correlation between the levels of BACE1 and full-length NRG1 precursor in the BA6 brain region of healthy control group. This positive relationship between BACE1 and NRG1 was not observed in the schizophrenic group; however, a reduction of the NRG1-CTF was observed in this brain region (Barakat et al., 2010). Using human postmortem brain tissue, an independent research group further found brain region-specific changes of NRG1 cleavage in schizophrenic patients with a great increase in the ratio of NRG1-NTF to full length NRG1 in the BA9 region (Marballi et al., 2012). However, to our knowledge, there is no study of BACE1-dependent NRG1 cleavage activity in living patients with schizophrenia.

#### Genetic Studies of *NRG1* in Schizophrenia

In contrast, the genetic studies of *NRG1* in schizophrenia have been extensively investigated. While most human genetic studies indicated that variants of *NRG1* might increase risk to psychiatric disorders including schizophrenia, there are still reports from various studies with controversial results.

*NRG1* was discovered as a prime candidate gene for schizophrenia by Stefansson et al. (2002) who used Systematic linkage disequilibrium (LD) mapping of 8p12–21 in an Icelandic study. Since then, other reports from different countries have been published, such as studies from Japan (Fukui et al., 2006), China (Yang et al., 2003), Scotland (Thomson et al., 2007), India (Kukshal et al., 2013), Italy (Squassina et al., 2010), Denmark (Ingason et al., 2006), Pakistan (Naz et al., 2011), Finland (Turunen et al., 2007), and Sweden (Alaerts et al., 2009). However, different haplotypes of NRG1 were found from various studies. For instance, in the Icelandic population, SNP haplotype in the 5′ region of *NRG1* (HAP_ICE_: SNP8NRG221533, SNP8NRG241930, SNP8NRG243177) was identified with linkage of schizophrenia risk (Stefansson et al., 2002), while in the Scottish population, a significant association between *NRG1* (HAP_ICE_) and schizophrenia was detected by PCR (Stefansson et al., 2003). In Japan, researchers failed to replicate the association between *NRG1* and schizophrenia in a large Japanese population, while no association between *NRG1* and schizophrenia was also reported in a large Danish sample (Ingason et al., 2006; Ikeda et al., 2008). In addition, a novel haplotype of the *NRG1* gene was found to confer risk of schizophrenia susceptibility in Chinese Han, but not in the Icelandic/Scottish population (Li et al., 2004). This suggests that stratification and phenotypic heterogeneity may have constrained detection of genetic associations. Other variations or haplotypes located in *NRG1* were also associated with schizophrenia using different SNPs tagging, analysis methods, sample size, and populations. Using association analysis method, one study showed variants in *NRG1* (rs2919381) and *ERBB4* might contribute to susceptibility to schizophrenia in Japanese population (Shiota et al., 2008). Evidence for *NRG3* (rs1937970 and rs677221) as a susceptibility gene for schizophrenia was identified in Chinese Han population (Wang Y.C. et al., 2008). Using LD method, the haplotype 221121 of *NRG1* and its six SNPs were associated to schizophrenia in Indian population (Kukshal et al., 2013). In a study of Northern Swedish Isolated Population, five SNPs located in the second intron of *NRG1* were found with schizophrenia association also by LD method (Alaerts et al., 2009). Variants of NRG1 can be detected genetic association with schizophrenia in different periods and features of patients, which can further confirm these risks to disease.

#### *NRG1* and Schizophrenia Clinical Categories

Schizophrenia symptoms are typically classified under four broad categories: positive symptoms, negative symptoms, disorganization, and cognitive dysfunction (van Os and Kapur, 2009). *NRG1* is considered as a risk gene for schizophrenia, and variants of it are associated with schizophrenia clinical symptoms. Bakker et al. (2004) divided schizophrenic patients into two groups based on their chronic idiopathic negative symptoms as deficit group (negative symptoms) and non-deficit group by the Schedule for the Deficit Syndrome (Carpenter et al., 1988). They found *NRG1* (SNP8NRG221533) was related to the non-deficit schizophrenia subtype only in Caucasian population (Bakker et al., 2004). Later, a study tested three SNPs (SNP8NRG 221132, SNP8NRG241930, and SNP8NRG 243177) in Hungarian population and found only SNP8NRG241930 was related to cognitive and hostility factors by PANSS in non-deficit schizophrenia (Rethelyi et al., 2010). Another case-control study in Caucasian population showed several haplotypic variants of *NRG1* (SNP8NRG221533 SNP8NRG241930 SNP8NRG243177 MS478B14-848 MS420M9-1395) had “protective” effects on age of onset and positive symptoms of schizophrenia (Papiol et al., 2011), which is consistent with the findings in other investigations (Kim et al., 2006; Alaerts et al., 2009). Recently, in a study of Iranian population, Yoosefee et al. (2016) found the G allele of rs2439272 might be significant association with negative symptoms especially in male participants and increased risk of developing schizophrenia.

#### *NRG1* and Neurophysiological Endophenotypes of Schizophrenia

Endophenotypes are thought to be more stable and homogenous than clinical syndromes. Neurophysiological endophenotypes of schizophrenia are characterized by a series of biological and behavioral traits, such as changes in cognitive function, PPI, EMT, ERP and neuroimaging (Braff and Light, 2005). For example, deficits of the inhibition function in patients with schizophrenia were suggested by many studies (Turetsky et al., 2007). The impaired inhibition function can be expressed as changes of PPI, ASEM, SPEM, P50 auditory evoked potential suppression, P300 event-related brain potential, and more. Here, we will discuss the relationship between *NRG1* gene and a few specific endophenotypes of schizophrenia.

##### *NRG1* and PPI

Pre-pulse inhibition is a neurological phenomenon that has been widely used for detecting inhibitory sensory motor gating of the startle reflex, and it is recognized as one of the schizophrenic endophenotypes (Cadenhead et al., 2000; Kumari et al., 2005). There have been several clinical studies that suggest a relationship between *NRG1* gene and PPI. One study demonstrated the lowest level of PPI in Caucasians and African Americans schizophrenia subjects who also carried the homozygous A allele (*NRG1* rs3924999) (Hong et al., 2008). Another study showed that carrying *NRG1* risk genotype variations (SNP8NRG241930, rs6994992, rs2439272 rs10503929 and rs3924999) was related to reduced PPI in healthy subjects (Roussos et al., 2011). These reports suggest that individuals with *NRG1* phenotype might be associated with attenuation of PPI, regardless of if they are healthy populations or patients with schizophrenia. While the underlying mechanisms involving *NRG1* genotype in PPI are unknown, studies implicated that Nrg1 regulates NMDA receptors in specific brain regions that could induce PPI reduction and contribute to schizophrenia-like symptoms (Javitt and Lindsley, 2001; Gu et al., 2005; Hahn et al., 2006). Thus, glutamate signaling may be a potential target for the relationship between *NRG1* and PPI.

##### *NRG1* and ERP

The brain’s gating function refers to the capacity to filter out duplicated or redundant stimuli (Freedman et al., 1996). ERP, the measurement of brain response to a specific sensory, cognitive, or motor event, is a schizophrenic endophenotype. Using electroencephalography, several waveforms have been found to be related to ERP, such as N100, P50, and P300 (Hall et al., 2007). While P300 reflects attentive resource allocation to the relevant stimulation, P50 sensory gating reflects the filtering process to irrelevant stimulus in the early stage of brain attentive function (Polich and Kok, 1995; Wan et al., 2008). Studies of patients with schizophrenia demonstrated that NRG1-induced AKT phosphorylation is associated with P50 suppression observed in first-episode patients with schizophrenia. This finding suggests that the PI3K/AKT system may be involved in the impaired sensory gating observed in schizophrenia (Keri et al., 2010). In concert with this finding, a study of acoustic startle response and P50 in patients with schizophrenia showed greater S2 response amplitude and deficit of P50 suppression in patients with schizophrenia than in controls. However, no correlations between PPI and P50 suppression were found in either patients with schizophrenia or control groups (Storozheva et al., 2016), suggesting different mechanisms underlie specific schizophrenia endophenotypes. Regarding investigation of the relationship between *NRG1* gene and ERP in schizophrenia, a study found a significant linkage between SNP8NRG221533 and P300 latency, showing individuals carrying more C alleles had greater P300 latency delay (Bramon et al., 2008). However, there was no significant association between *NRG1* SNPs (SNP8NRG221533, SNP8NRG241930, and SNP8NRG243177) and P50 suppression observed in a large schizophrenia endophenotype study (Shaikh et al., 2011). A recent meta-analytic review concluded that P50 suppression, P300 amplitude, and P300 latency may serve as viable endophenotypes for schizophrenia (Earls et al., 2016). Therefore, whether *NRG1* is related to specific schizophrenic endophenotypes might need further investigations.

##### *NRG1* and eye movement deficits

Eye movement deficits, particularly in SPEM and ASEM, are important endophenotypes in patients with schizophrenia (Meyhofer et al., 2015; Wan et al., 2017). While a number of studies demonstrated 50–80% of patients with schizophrenia have impaired SPEM compared to 8% of healthy individuals (Lencer et al., 2003; Ettinger et al., 2004), few studies have investigated genetic association of eye movement deficits with *NRG1*. A study of *NRG1* genotypes with eye movement deficits in 113 patients with schizophrenia and 106 age-matched healthy controls found no relationship between *NRG1* genotype (SNP8NRG222662, SNP8NRG243177) and ASEM or SPEM task performance (Haraldsson et al., 2010). Consistent with Haraldsson’s study, two studies in Korea also found no associations between *NRG1* (rs35753505G, rs4623364G; rs6994992T rs3924999A) and ASEM or SPEM abnormality (Pasaje et al., 2011; Kim et al., 2012). However, the result in healthy subjects showed interaction between *NRG1* and eye movement deficits. One study found SNP8NRG243177 in healthy young males was related to SPEM by using the root-mean-square error method (Smyrnis et al., 2011), while another study showed a significant effect of *NRG1* rs3924999 genotype on ASEM amplitude gain, but not to SPEM or other variables of ASEM, in 114 healthy Caucasian subjects (Schmechtig et al., 2010), suggesting *NRG1* genotypes may affect visuospatial sensorimotor transformations in general and could be a potential mechanism underlying impaired eye movements in patients with schizophrenia.

##### *NRG1* and neuropathology

Some of the major schizophrenia pathological characters are brain atrophy (Harvey et al., 1993; Lim et al., 1996), reduction of whole brain volumes (Gaser et al., 2004), and abnormality in density as well as integrity in diverse brain areas (Burns et al., 2003; Kubicki et al., 2003; Sun et al., 2003; Wang et al., 2004). As NRG1 plays critical roles in myelination, there is an increase in an attention to the *NRG1* gene variant association with neuropathology in patients with schizophrenia.

A reduction of white matter density and integrity in the ALIC and prefrontal subgyrus in *NRG1* (SNP8NRG243177) carriers was first reported in 2008 (McIntosh et al., 2008), while the SNP8NRG221533 genotype of *NRG1* was reported as affecting medial frontal white matter microstructure (Winterer et al., 2008). Later, studies on SNP8NRG221533 in schizophrenia showed that the *NRG1* variation was related to decreased anterior cingulum fractional anisotropy (Wang et al., 2009), lower volume of internal capsule (Cannon et al., 2012), and reduced volume of left UF (Voineskos et al., 2013). In addition to white matter, studies also explored the effect of *NRG1* variation on gray matter volume. For example, two studies suggested that *NRG1* (rs35753505) was significantly associated with gray matter volume reduction (Knickmeyer et al., 2014; Thirunavukkarasu et al., 2014), while another investigation found a significant association between SNP8NRG222662 (rs4623364) and reduced volume of left superior temporal gyrus cortex (Tosato et al., 2012). However, whether *NRG1* genetic variations directly cause brain structural and functional changes in schizophrenia remains unclear and further studies in schizophrenic patients with neuroimaging in combination with other disease-specific biomarkers would be helpful.

In summary, human studies demonstrated that *NRG1* as a schizophrenia-linked candidate gene plays an important role in the pathological process of schizophrenia through its effect on brain function. Together, findings provide evidence to support an important role of NRG1 in neurodevelopment and susceptibility to schizophrenia (**Table 1**).

**Table 1:** Effect of *NRG1* on schizophrenia in human studies.

	Features	Results	Reference
Protein expression in the brain	N-terminal	The level of NRG1-NTF was increased in BA9 of schizophrenia	Marballi et al., 2012
	C-terminal	The level of NRG1-CTF was decreased in BA6 of schizophrenia	Barakat et al., 2010
	Full length	The level of full-length NRG1 was lower in BA9 of schizophrenia; No changes in BA6 of schizophrenia	Barakat et al., 2010; Marballi et al., 2012
Genetic association	HAP_ICE_	SNP8NRG221533, SNP8NRG241930, SNP8NRG243177 was reported positive association in Scottish population, negative association in Japanese population, Danish population, Chinese population	Stefansson et al., 2003; Li et al., 2004; Ingason et al., 2006; Ikeda et al., 2008
	Novel haplotypes and SNPs	The haplotype 221121 (rs35753505-rs6994992-rs1354336-rs10093107-rs3924999-rs11780123) in India population; Rs7017348, rs6468061, rs7014221, rs7014410, rs17601950 in northern Swedish Isolated population; Rs2919381 in Japanese population; HAP_China__1_, HAP_China__2_ and HAP_China__3_ in Chinese population	Li et al., 2004; Shiota et al., 2008; Alaerts et al., 2009; Kukshal et al., 2013
Clinical Categories	Non-Deficit	SNP8NRG241930 in Hungary population; SNP8NRG221533 in Caucasian population	Bakker et al., 2004; Rethelyi et al., 2010
	Protective effect	SNP8NRG221533, SNP8NRG241930, SNP8NRG243177, MS478B14-848, MS420M9-1395 in Caucasian population	Papiol et al., 2011
	Negative symptom	Rs2439272 in Iranian population	Yoosefee et al., 2016
Endophenotypes	PPI	SNP8NRG241930, rs6994992, rs2439272 rs10503929 and rs3924999 in Greek healthy males; Rs3924999 in Caucasians and African Americans	Hong et al., 2008; Roussos et al., 2011
	ERP	SNP8NRG221533 in Maudsley Family	Bramon et al., 2008
	EMT	SNP8NRG243177 related to SPEM in healthy young males; Rs3924999 related to ASEM in healthy Caucasian	Schmechtig et al., 2010; Smyrnis et al., 2011
	Neuroimaging	White matter: SNP8NRG243177 related to reduction white matter in ALIC and prefrontal subgyrus; SNP8NRG221533 related to medial frontal white matter microstructure; decreased anterior cingulum fractional anisotropy; lower volume of internal capsule; lower volume of left UF Gray matter: Rs35753505 related to gray matter volume reduction; SNP8NRG222662 related to lower volumes of left superior temporal gyrus cortex	McIntosh et al., 2008; Winterer et al., 2008; Wang et al., 2009; Cannon et al., 2012; Tosato et al., 2012; Voineskos et al., 2013; Knickmeyer et al., 2014; Thirunavukkarasu et al., 2014

*BA, Brodmann’s Area; HAP, Haplotypes; SNP, Single Nucleotide Polymorphism; PPI, Pre-Pulse Inhibition; ERP, Event-Related Potential; EMT, Eye Movement Test; SPEM, Smooth Pursuit Eye Movements; ASEM, Anti-Saccade Eye Movements; ALIC, Anterior Limb of the Internal Capsule; UF, Uncinate Fasciculus.*

### Preclinical Research: *Nrg1* and *BACE1* Gene Modified Animal Models

While human studies have demonstrated that NRG1 plays critical roles in schizophrenia, preclinical research using gene knockout or mutant mice have provided some valuable evidence of association between Bace1 and Bace1-Nrg1 cleavage and schizophrenia by behavioral studies as well as pharmacological investigations.

#### Mice with Mutated *Nrg1* Develop Schizophrenia-Like Behaviors

During the last decades, several types of Nrg1 transgenic mice have been developed to explore the effect of Nrg1 on behaviors, as well as the underlying mechanisms. One of which is a mouse model of heterozygous transmembrane domain Nrg1 mutant (TM-*Nrg1*^+/-^). The TM-*Nrg1*^+/-^ mice develop dysfunctional NMDA receptors in the forebrain, impaired PPI, and increased spontaneous activity that clozapine treatment was able to reverse (Stefansson et al., 2002). Another feature of TM-*Nrg1*^+/-^ mice was age- and brain region-related alternations of NMDA and D_2_ receptor levels which cause selective disturbance of glutamatergic and dopaminergic neurotransmission in the animals (Newell et al., 2013). A mouse model with a different mutation of *Nrg1*, a heterozygous mutation in Nrg1 immunoglobulin-like domain (Ig*-Nrg1*^+/-^), displayed schizophrenia-like behaviors, particularly suppression of open field, running wheel, and T-maze. The Ig*-Nrg1* ^+/-^ mice were more sensitive to clozapine treatment (Rimer et al., 2005). Additionally, animal models of overexpression with different Nrg1 isoforms also developed schizophrenia-liked behaviors. For example, 11-month-old mice with overexpression of *Nrg1-type I* showed impaired hippocampal-dependent spatial working memory and oscillations (Deakin et al., 2012), while *Nrg1-type III*-overexpressed transgenic mice developed sensorimotor gating deficits with changes in the activity of circuit projections from the vHPC to the nACC (Nason et al., 2011). Disrupted cortical-amygdala neural circuits have also been observed in similar transgenic mice, leading to altered processing of salient memories (Jiang et al., 2013). A novel transgenic mouse model of overexpressed *Nrg1-type IV* (Nrg1-IV/NSE-tTA) also exhibited impaired sensorimotor function, discrimination memory, and social behaviors. The Nrg1-IV/NSE-tTA mice also expressed disrupted dendritic development, synaptic pathology, and excitatory-inhibitory imbalance in the prefrontal cortex, which may be mediated by ErbB4 and the downstream signal target, PIK3-p110δ (Papaleo et al., 2016). Interestingly, overexpression of secreted Nrg1 by Bace1 cleavage (Nrg1-ntf_β_) in mice was sufficient to cause schizophrenia-like phenotypes. The abnormal behaviors were Nrg1-ntf_β_-specific since turning off the Nrg1-ntf_β_ expression genetically can reverse the schizophrenia-like behaviors in the mouse model (Luo et al., 2014). Lines of evidence suggested that gain-off function mutations in Nrg1 are also risk factors for schizophrenia. According to these *Nrg1* genetic models, it is possible that dysfunction of NRG1 or NRG1/ErbB4 signaling may affect neural development and synaptic plasticity by disturbance of glutamatergic or GABAergic systems implicated in schizophrenia. We therefore summarized that schizophrenia-like behaviors are related to various *Nrg1* mutations (**Table 2**).

**Table 2:** The effect of *Nrgl* and *Bacel* mutation on schizophrenia-like genotypes in mice.

Genotyping	Feature of mice	Behaviors	Pathology	Reference
*TM-Nrg1*^+/-^	Transmembrane region deletion in heterozygous mice	Impaired PPI, increased spontaneous activity	Fewer NMD A receptor level ; Disturbance glutamatergic and dopaminergic neurotransmission in different ages	Stefansson et al., 2002; Newell et al., 2013
*Ig-Nrg1*^+/-^	Mutation in *Nrg1* immunoglobulin-like domain in heterozygous mice	Reduced activity in open field, running wheel and T-maze, decreased latent inhibition with clozapine treatment		Rimer et al., 2005
Overexpressing *Nrg1-type I*	*Nrg1-type I* overexpressed in 11-month-old mice	Impaired spatial working memory	Altered hippocampal oscillatory, lower carbachol-induced epileptiform activity	Deakin et al., 2012
Overexpressing *Nrg1-type III*	*Nrg1-type III* overexpressed in mice	Sensorimotor gating deficits; Altered salient memories	Disrupted from vHPC to nACC circuit projections; Disrupted cortical-amygdala neural circuits	Nason et al., 2011; Jiang et al., 2013
Overexpressing *Nrg1-I VI*NSE-tTA	Selectively *Nrg1-W* overexpressed in a neuronal specific manner mice	Impaired sensorimotor, discrimination memory and social behaviors	Abnormal synaptic, imbalance ex-inhibitory in PFC	Papaleo et al., 2016
Overexpressing *Nrg1-ntfβ*	N-terminal fragment overexpressed in mice	Reduced spontaneous alternations, impaired contextual fear conditioning	Deceased NMDA receptors	Luo et al., 2014
*Bacel*^-/-^	*Bace1* gene knock out mice	Impaired PPI, working memory and social recognition; Spontaneous hyperactivity	Accumulation of intact Nrgl; Impaired process of myelination; Disturbed NRGl/ErbB4 signaling pathway; Disturbed NRG1/AKT signaling pathway	Hu et al., 2006, 2008; Willem et al., 2006; Savonenko et al., 2008; Seshadri et al., 2010

*TM, Transmembrane; Ig, Immunoglobulin; vHPC, Ventral Hippocampus; nACC, Nucleus Accumbens; PFC, Prefrontal Cortex.*

#### Mutation of Bace1 Mice Show Schizophrenia-Like Behaviors

As a transmembrane protease, BACE1 is important for several disease-related substrates, including beta amyloid peptide production in AD and NRG1 in schizophrenia (Wang et al., 2013). In addition to BACE1 cleavage of a series of types of Nrg, including Nrg1-type I, Nrg1-type III, and Nrg3, BACE1 also cleaves the β2 subunit of voltage-gated sodium channels (Na_v_1, β2) (Corbett et al., 2013) that participate in regulation of neuronal development and maintenance of normal brain function. Studies of *Bace1*^-/-^ mice showed reduction of myelination, deficits in cognitive performance, and impaired emotional activity (Harrison et al., 2003; Hu et al., 2006). Moreover, the *Bace1*^-/-^ mice showed seizure-like genotype with increased expression of Na_v_1β2 in hippocampal areas, which is related to hyperactivity and elevated excitability of hippocampal neurons (Hu et al., 2010). Together, results suggest the possible relationship between BACE1 and dysfunctions of the brain such as schizophrenia, epileptic seizures, and AD.

Whether there are any specific effects of Bace1 cleavage of Nrg1 on animal behavior is still in question. Several studies of *Bace1* knockout mice have found reduction of Nrg1-type I and type III β1 levels, elevated full length Nrg1, and diminished activation of Akt in the brain (Willem et al., 2006), along with a delayed process of myelination and reduced myelin thickness (Hu et al., 2006, 2008). This suggests that BACE1-dependent cleavage of Nrg1 may regulate myelination and myelin sheath thickness by mediating phosphorylation of Akt. As myelin and oligodendrocyte function could affect neuronal connectivity, the dysfunction of myelination may well be related to the neuropathogenesis of schizophrenia (Nave and Ehrenreich, 2014). Additionally, the *Bace1*^-/-^ mice treated with a glutamatergic psychostimulant showed impaired PPI, working memory, and social recognition, as well as spontaneous hyperactivity as schizophrenia-like behaviors. Decreased spine density in hippocampal pyramidal neurons was also observed in *Bace1*^-/-^ mice via NRG1/ErbB4 signal pathway regulation (Savonenko et al., 2008), suggesting that disturbed NRG1/ErbB4 signaling pathways in the *Bace1*^-/-^ mouse model may contribute to the pathophysiology of schizophrenia. There was a decreased DISC1 expression reported in *Nrg1*^-/-^ knockout mice, as well as in *Bace1*^-/-^ mice, which might be linked to impaired NRG1/AKT signal pathway (Seshadri et al., 2010). As described above, animal studies suggest that BACE1 might be involved in the pathology of schizophrenia via cleaving substrates to stimulate the downstream signal pathway (**Table 2**).

#### Nrg1 and Antipsychotic Treatment

The mechanism of antipsychotics is complicated, and includes binding with DA, 5-HT, H1, M1, and α receptors. In addition, some antipsychotics are selective for specific symptoms. For instance, risperidone works better on positive symptoms while others like aripiprazole can improve the severity of negative symptoms (Komossa et al., 2011; Maher and Theodore, 2012). While many studies focus on the effect of antipsychotic treatment on the alteration of *NRG1* gene expression in animal models, there are few human reports in this field due to the ethical issues and method limitation.

A clinical study in Chinese Han patients indicated that exposure to risperidone and quetiapine for 4 weeks could increase the NRG1 expression of peripheral blood lymphocytes of first episode schizophrenia (Zhang et al., 2008). Another study showed that clozapine treatment elevated *NRG1* expression in human fetal brain aggregates, which was not yet observed in a haloperidol-treated group (Chana et al., 2009). These human studies suggest that different antipsychotic treatments may cause differential effects on expression of *NRG1*. Results from animal studies also indicate that the duration of antipsychotics also contributed to various changes of *Nrg1*. The levels of Nrg1 and ErbB4 receptors in rat prefrontal cortex and hippocampus were increased by treatment with haloperidol for 4 weeks (Wang X.D. et al., 2008), while an 8-week haloperidol treatment showed no effect on Nrg1 levels in mice (Shibuya et al., 2010). Furthermore, a 12-week haloperidol treatment experiment reduced the ErbB4 activation (Hahn et al., 2006), as well as expression of Nrg1 and ErbB4, in the brains of mice (Pan et al., 2011). Overall, these studies suggest that not only type of antipsychotics, but also duration of antipsychotic treatment, may be a crucial factor to change *Nrg1* expression, while also considering the brain region-specific effects of antipsychotics (**Table 3**).

**Table 3:** Effects of antipsychotic drugs on expression of Nrg1 and ErbB4 signaling.

	Subjects	Drugs	Dosage	Treatment duration	Nrg1/ErbB4	Reference
Human studies	PBL cells	Clozapine/ Haloperidol	2 μM/500 nM	3 weeks	Up/No changes	Chana et al., 2009
	Onset patients	Risperidone/ Quetiapine	(533.33±71.45)mg/ day/(544.62±63.85) mg/day	4 weeks	Up/Up	Zhang et al., 2008
Animal studies	Rat	Haloperidol/ Risperidone/ Clozapine	1 mg/kg i.p./1 mg/kg i.p./ 10 mg/kg i.p.	4 weeks	Up/Up/Down	Wang Y.C. et al., 2008
	Monkey	Haloperidol	0.125–0.25 mg/mL/day	8 weeks	No changes	Shibuya et al., 2010
	Mice	Haloperidol	2 mg/kg/day	12 weeks	Down	Hahn et al., 2006
	Rat	Aripiprazole/ Olanzapine/ Haloperidol	UN	12 weeks	Down/Down/Down	Pan et al., 2011

*Up, Up Regulation; Down, Down Regulation; PBL, Peripheral Blood Lymphocytes; UN, Unknown.*

BACE1 inhibitor as a therapeutic strategy to improve cognitive in AD has been challenging. Both safety and efficacy are questionable. *In vitro*, inhibition of BACE1 can cause adverse side effects during synaptic developmental stages (Kamikubo et al., 2017). However, there are almost no reports on psychotic symptoms from BACE1 inhibitor clinical trials rather than improved cognitive function in AD patients (Kennedy et al., 2016; Timmers et al., 2017). We speculated that the current available BACE1 inhibitors might be made for targeting on APP which has different cleavage site than other substrates as NRG1. Further investigations on substrate-dependent BACE1 cleavage activity are needed.

In the future, exploring the dynamic changes of BACE1-dependent NRG1 cleavage process in biological samples from schizophrenic patients would be important. It will provide new insights into how BACE1-dependent NRG1 proteolytic processing could contribute to the pathophysiology of schizophrenia, and help to discover the underlying biomarker of schizophrenia, which is essential for early diagnosis of the disorder disease and effective medical treatment.

## Concluding Remarks

**FIGURE 3:**
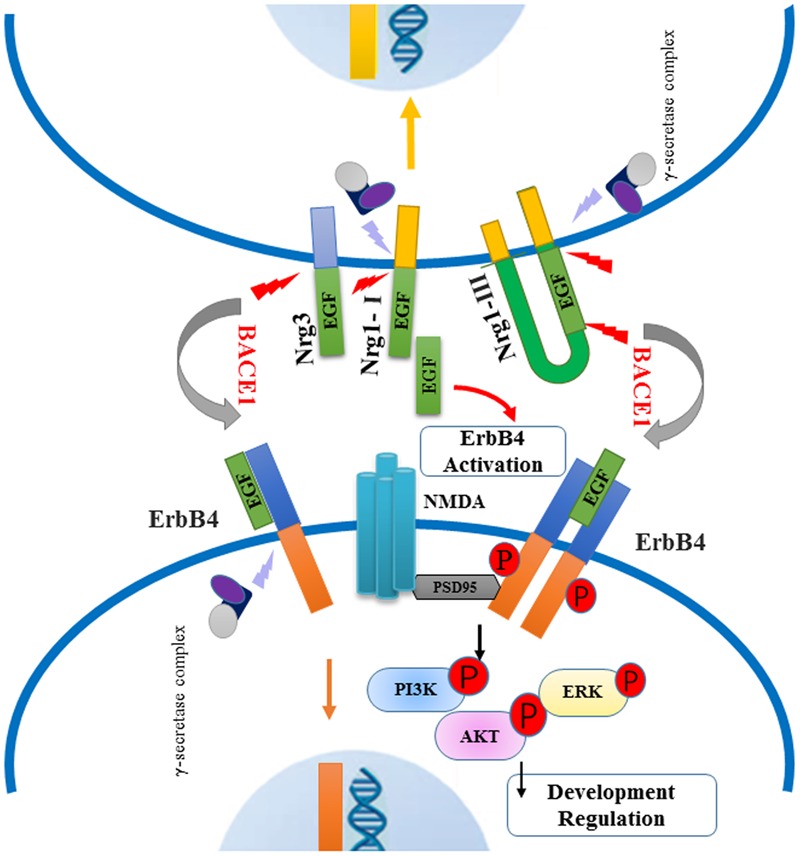
Schematic of BACE1-dependent NRG1/ErbB4 signaling pathway involving in the pathogenies of schizophrenia. Neuregulins (type I, type III Nrg1 and Nrg3) are cleaved by BACE1 and release their EGF-domain into in the extracellular space, through binding the ErbB4 receptors to activate downstream signaling pathway. The NRG1/ErbB4 signaling in neurons can exert an effect on NMDA receptors interacting with PSD-95, which lead to the phosphorylation of PI3K-AKT and ERK molecules. Abnormal NRG1/ErbB4 signaling pathway may contribute to impaired myelination and synaptic function. Meanwhile, intracellular fragment of Nrg1 and ErbB4 are cut off by γ-secretase complex, producing the peptide into the nuclear to regulate neuron development.

Neuregulin, especially Nrg1, plays a major role as the psychological substrate of BACE1. Numerous lines of evidence support the hypothesis that Nrg1 can contribute to the pathophysiology of schizophrenia. Both, human and animal research, suggest that BACE1-dependent Nrg1 cleavage and NRG1/ErbB4 signaling may play specific roles in schizophrenia, as summarized in **Figure 3**. Several BACE1 inhibitors have entered into phase I studies, and at least one of these inhibitors has advanced to phase III human trails. Due to various BACE1 substrates, it is helpful to investigate their role and further illustrate the function of Nrg1 downstream signaling pathways in schizophrenia. It is important for understanding the biological mechanism of BACE1 together with its substrates Nrg1, and further exploring effective and specific inhibitor drugs for schizophrenia, not interfering other biological progress, which could provide possible therapeutic strategies for this psychiatry disorder. In future studies, it will be important to investigate BACE1, Nrg1-related molecular pathways, and neural circuits in endophenotypes resembling features of schizophrenia.

## Author Contributions

RL: Initiated research topic and discussed literatures and hypothesis within the review topic. Some editing as well. ZZ: Wrote major part of the review. JH: Wrote some part of the review. YS: Edited manuscript.

## Conflict of Interest Statement

The authors declare that the research was conducted in the absence of any commercial or financial relationships that could be construed as a potential conflict of interest.

## Abbreviations

AD: Alzheimer Disease
AKT: serine/threonine kinase 1
ALIC: anterior limb of the internal capsule
ASEM: anti-saccade eye movements
BACE1: β-secretase
DISC1: disrupted in schizophrenia 1
EGF: epidermal growth factor
EMT: eye movement test
ErbB4: erb-b2 receptor tyrosine kinase 4
Erk: extracellular regulated MAP kinase
ERP: event-related potential
HAP: haplotype
Ig: immunoglobulin
nACC: nucleus accumbens
NMDA: N-methyl-D-aspartic acid
NRG1: Neuregulin-1
NRG1-CRD: NRG1-intracellular domain
Nrg1-ctf: Nrg1 C-terminal fragment
Nrg1-ntf: Nrg1 N-terminal fragment
NRG3: Neuregulin-3
PI3K: phosphatidylinositol-4,5-bisphosphate 3-kinase
PPI: pre-pulse inhibition
PSD95: postsynaptic density protein 95
SPEM: smooth pursuit eye movements
TACE: tumor necrosis factor-α-converting enzyme
UF: uncinate fasciculus
vHPC: ventral hippocampus

## References

[B1] Alaerts M. Ceulemans S. Forero D. Moens L. N. De Zutter S. Heyrman L. (2009). Support for NRG1 as a susceptibility factor for schizophrenia in a northern Swedish isolated population. *Arch. Gen. Psychiatry* 66 828–837. 10.1001/archgenpsychiatry.2009.82 19652122

[B2] Bakker S. C. Hoogendoorn M. L. Selten J. P. Verduijn W. Pearson P. L. Sinke R. J. (2004). Neuregulin 1: genetic support for schizophrenia subtypes. *Mol. Psychiatry* 9 1061–1063. 10.1038/sj.mp.4001564 15303101

[B3] Bao J. Lin H. Ouyang Y. Lei D. Osman A. Kim T. W. (2004). Activity-dependent transcription regulation of PSD-95 by neuregulin-1 and Eos. *Nat. Neurosci.* 7 1250–1258. 10.1038/nn1342 15494726

[B4] Barakat A. Dean B. Scarr E. Evin G. (2010). Decreased Neuregulin 1 C-terminal fragment in Brodmann’s area 6 of patients with schizophrenia. *Schizophr. Res.* 124 200–207. 10.1016/j.schres.2010.09.001 20926259

[B5] Bartolini G. Sanchez-Alcaniz J. A. Osorio C. Valiente M. Garcia-Frigola C. Marin O. (2017). Neuregulin 3 mediates cortical plate invasion and laminar allocation of GABAergic interneurons. *Cell Rep.* 18 1157–1170. 10.1016/j.celrep.2016.12.089 28147272 PMC5300889

[B6] Benzel I. Bansal A. Browning B. L. Galwey N. W. Maycox P. R. McGinnis R. (2007). Interactions among genes in the ErbB-Neuregulin signalling network are associated with increased susceptibility to schizophrenia. *Behav. Brain Funct.* 3:31 10.1186/1744-9081-3-31 PMC1934910 17598910

[B7] Boerboom A. Dion V. Chariot A. Franzen R. (2017). Molecular mechanisms involved in Schwann cell plasticity. *Front. Mol. Neurosci.* 10:38 10.3389/fnmol.2017.00038 PMC5314106 28261057

[B8] Braff D. L. Light G. A. (2005). The use of neurophysiological endophenotypes to understand the genetic basis of schizophrenia. *Dialogues Clin. Neurosci.* 7 125–135. 16262208 10.31887/DCNS.2005.7.2/dlbraff PMC3181726

[B9] Bramon E. Dempster E. Frangou S. Shaikh M. Walshe M. Filbey F. M. (2008). Neuregulin-1 and the P300 waveform–a preliminary association study using a psychosis endophenotype. *Schizophr. Res.* 103 178–185. 10.1016/j.schres.2008.03.025 18571900

[B10] Bublil E. M. Yarden Y. (2007). The EGF receptor family: spearheading a merger of signaling and therapeutics. *Curr. Opin. Cell Biol.* 19 124–134. 10.1016/j.ceb.2007.02.008 17314037

[B11] Burgess T. L. Ross S. L. Qian Y. X. Brankow D. Hu S. (1995). Biosynthetic processing of neu differentiation factor. Glycosylation trafficking, and regulated cleavage from the cell surface. *J. Biol. Chem.* 270 19188–19196. 10.1074/jbc.270.32.19188 7642587

[B12] Burns J. Job D. Bastin M. E. Whalley H. Macgillivray T. Johnstone E. C. (2003). Structural disconnectivity in schizophrenia: a diffusion tensor magnetic resonance imaging study. *Br. J. Psychiatry* 182 439–443. 10.1192/bjp.182.5.439 12724248

[B13] Cadenhead K. S. Swerdlow N. R. Shafer K. M. Diaz M. Braff D. L. (2000). Modulation of the startle response and startle laterality in relatives of schizophrenic patients and in subjects with schizotypal personality disorder: evidence of inhibitory deficits. *Am. J. Psychiatry* 157 1660–1668. 10.1176/appi.ajp.157.10.1660 11007721

[B14] Cannon D. M. Walshe M. Dempster E. Collier D. A. Marshall N. Bramon E. (2012). The association of white matter volume in psychotic disorders with genotypic variation in *NRG1, MOG* and *CNP*: a voxel-based analysis in affected individuals and their unaffected relatives. *Transl. Psychiatry* 2 e167. 10.1038/tp.2012.82 PMC3565820 23032943

[B15] Carpenter W. T. Jr. Heinrichs D. W. Wagman A. M. (1988). Deficit and nondeficit forms of schizophrenia: the concept. *Am. J. Psychiatry* 145 578–583. 10.1176/ajp.145.5.578 3358462

[B16] Chana G. Lucero G. Salaria S. Lozach J. Du P. Woelk C. (2009). Upregulation of NRG-1 and VAMP-1 in human brain aggregates exposed to clozapine. *Schizophr. Res.* 113 273–276. 10.1016/j.schres.2009.05.015 19502011 PMC2759675

[B17] Chavarria-Siles I. White T. de Leeuw C. Goudriaan A. Lips E. Ehrlich S. (2016). Myelination-related genes are associated with decreased white matter integrity in schizophrenia. *Eur. J. Hum. Genet.* 24 381–386. 10.1038/ejhg.2015.120 26014434 PMC4757770

[B18] Chen S. Velardez M. O. Warot X. Yu Z. X. Miller S. J. Cros D. (2006). Neuregulin 1-erbB signaling is necessary for normal myelination and sensory function. *J. Neurosci.* 26 3079–3086. 10.1523/JNEUROSCI.3785-05.2006 16554459 PMC6674097

[B19] Chen Y. Hancock M. L. Role L. W. Talmage D. A. (2010). Intramembranous valine linked to schizophrenia is required for neuregulin 1 regulation of the morphological development of cortical neurons. *J. Neurosci.* 30 9199–9208. 10.1523/JNEUROSCI.0605-10.2010 20610754 PMC2919805

[B20] Corbett B. F. Leiser S. C. Ling H. P. Nagy R. Breysse N. Zhang X. (2013). Sodium channel cleavage is associated with aberrant neuronal activity and cognitive deficits in a mouse model of Alzheimer’s disease. *J. Neurosci.* 33 7020–7026. 10.1523/JNEUROSCI.2325-12.2013 23595759 PMC6618875

[B21] Deakin I. H. Nissen W. Law A. J. Lane T. Kanso R. Schwab M. H. (2012). Transgenic overexpression of the type I isoform of neuregulin 1 affects working memory and hippocampal oscillations but not long-term potentiation. *Cereb. Cortex* 22 1520–1529. 10.1093/cercor/bhr223 21878485 PMC3377963

[B22] Dean B. Soulby A. Evin G. M. Scarr E. (2008). Levels of [(3)H]pirenzepine binding in Brodmann’s area 6 from subjects with schizophrenia is not associated with changes in the transcription factor SP1 or BACE1. *Schizophr. Res.* 106 229–236. 10.1016/j.schres.2008.08.003 18790604

[B23] Diez A. Cieza-Borrella C. Suazo V. Gonzalez-Sarmiento R. Papiol S. Molina V. (2014). Cognitive outcome and gamma noise power unrelated to neuregulin 1 and 3 variation in schizophrenia. *Ann. Gen. Psychiatry* 13 18 10.1186/1744-859X-13-18 PMC4065086 24976857

[B24] Earls H. A. Curran T. Mittal V. (2016). A meta-analytic review of auditory event-related potential components as endophenotypes for schizophrenia: perspectives from first-degree relatives. *Schizophr. Bull.* 42 1504–1516. 10.1093/schbul/sbw047 27217271 PMC5049529

[B25] Ettinger U. Kumari V. Crawford T. J. Corr P. J. Das M. Zachariah E. (2004). Smooth pursuit and antisaccade eye movements in siblings discordant for schizophrenia. *J. Psychiatr. Res.* 38 177–184. 10.1016/S0022-3956(03)00105-5 14757332

[B26] Falls D. L. (2003). Neuregulins: functions, forms, and signaling strategies. *Exp. Cell Res.* 284 14–30. 10.1016/S0014-4827(02)00102-7 12648463

[B27] Fernandez P. A. Tang D. G. Cheng L. Prochiantz A. Mudge A. W. Raff M. C. (2000). Evidence that axon-derived neuregulin promotes oligodendrocyte survival in the developing rat optic nerve. *Neuron* 28 81–90. 10.1016/S0896-6273(00)00087-8 11086985

[B28] Flores A. I. Mallon B. S. Matsui T. Ogawa W. Rosenzweig A. Okamoto T. (2000). Akt-mediated survival of oligodendrocytes induced by neuregulins. *J. Neurosci.* 20 7622–7630. 11027222 10.1523/JNEUROSCI.20-20-07622.2000 PMC6772890

[B29] Freedman R. Adler L. E. Myles-Worsley M. Nagamoto H. T. Miller C. Kisley M. (1996). Inhibitory gating of an evoked response to repeated auditory stimuli in schizophrenic and normal subjects. Human recordings, computer simulation, and an animal model. *Arch. Gen. Psychiatry* 53 1114–1121. 10.1001/archpsyc.1996.01830120052009 8956677

[B30] Fukui N. Muratake T. Kaneko N. Amagane H. Someya T. (2006). Supportive evidence for neuregulin 1 as a susceptibility gene for schizophrenia in a Japanese population. *Neurosci. Lett.* 396 117–120. 10.1016/j.neulet.2005.11.015 16326006

[B31] Gaser C. Nenadic I. Buchsbaum B. R. Hazlett E. A. Buchsbaum M. S. (2004). Ventricular enlargement in schizophrenia related to volume reduction of the thalamus, striatum, and superior temporal cortex. *Am. J. Psychiatry* 161 154–156. 10.1176/appi.ajp.161.1.154 14702264

[B32] Ghashghaei H. T. Weber J. Pevny L. Schmid R. Schwab M. H. Lloyd K. C. (2006). The role of neuregulin-ErbB4 interactions on the proliferation and organization of cells in the subventricular zone. *Proc. Natl. Acad. Sci. U.S.A.* 103 1930–1935. 10.1073/pnas.0510410103 16446434 PMC1413654

[B33] Gu Z. Jiang Q. Fu A. K. Ip N. Y. Yan Z. (2005). Regulation of NMDA receptors by neuregulin signaling in prefrontal cortex. *J. Neurosci.* 25 4974–4984. 10.1523/JNEUROSCI.1086-05.2005 15901778 PMC6724849

[B34] Hahn C. G. Wang H. Y. Cho D. S. Talbot K. Gur R. E. Berrettini W. H. (2006). Altered neuregulin 1-erbB4 signaling contributes to NMDA receptor hypofunction in schizophrenia. *Nat. Med.* 12 824–828. 10.1038/nm1418 16767099

[B35] Hall M. H. Rijsdijk F. Picchioni M. Schulze K. Ettinger U. Toulopoulou T. (2007). Substantial shared genetic influences on schizophrenia and event-related potentials. *Am. J. Psychiatry* 164 804–812. 10.1176/ajp.2007.164.5.804 17475740

[B36] Haraldsson H. M. Ettinger U. Magnusdottir B. B. Ingason A. Hutton S. B. Sigmundsson T. (2010). Neuregulin-1 genotypes and eye movements in schizophrenia. *Eur. Arch. Psychiatry Clin. Neurosci.* 260 77–85. 10.1007/s00406-009-0032-2 19575259

[B37] Harrison P. J. Law A. J. (2006). Neuregulin 1 and schizophrenia: genetics, gene expression, and neurobiology. *Biol. Psychiatry* 60 132–140. 10.1016/j.biopsych.2005.11.002 16442083

[B38] Harrison S. M. Harper A. J. Hawkins J. Duddy G. Grau E. Pugh P. L. (2003). BACE1 (beta-secretase) transgenic and knockout mice: identification of neurochemical deficits and behavioral changes. *Mol. Cell. Neurosci.* 24 646–655. 10.1016/S1044-7431(03)00227-6 14664815

[B39] Harvey I. Ron M. A. Du Boulay G. Wicks D. Lewis S. W. Murray R. M. (1993). Reduction of cortical volume in schizophrenia on magnetic resonance imaging. *Psychol. Med.* 23 591–604. 10.1017/S003329170002537X 8234567

[B40] Hashimoto R. Straub R. E. Weickert C. S. Hyde T. M. Kleinman J. E. Weinberger D. R. (2004). Expression analysis of neuregulin-1 in the dorsolateral prefrontal cortex in schizophrenia. *Mol. Psychiatry* 9 299–307. 10.1038/sj.mp.4001434 14569272

[B41] Hayes L. N. Shevelkin A. Zeledon M. Steel G. Chen P. L. Obie C. (2016). Neuregulin 3 knockout mice exhibit behaviors consistent with psychotic disorders. *Mol. Neuropsychiatry* 2 79–87. 10.1159/000445836 27606322 PMC4996025

[B42] Hong L. E. Wonodi I. Stine O. C. Mitchell B. D. Thaker G. K. (2008). Evidence of missense mutations on the neuregulin 1 gene affecting function of prepulse inhibition. *Biol. Psychiatry* 63 17–23. 10.1016/j.biopsych.2007.05.011 17631867 PMC3569848

[B43] Horiuchi K. Zhou H. M. Kelly K. Manova K. Blobel C. P. (2005). Evaluation of the contributions of ADAMs 9,12,15,17, and 19 to heart development and ectodomain shedding of neuregulins beta1 and beta2. *Dev. Biol.* 283 459–471. 10.1016/j.ydbio.2005.05.004 15936750

[B44] Hu X. He W. Diaconu C. Tang X. Kidd G. J. Macklin W. B. (2008). Genetic deletion of BACE1 in mice affects remyelination of sciatic nerves. *FASEB J.* 22 2970–2980. 10.1096/fj.08-106666 18413858 PMC2493455

[B45] Hu X. Hicks C. W. He W. Wong P. Macklin W. B. Trapp B. D. (2006). Bace1 modulates myelination in the central and peripheral nervous system. *Nat. Neurosci.* 9 1520–1525. 10.1038/nn1797 17099708

[B46] Hu X. Zhou X. He W. Yang J. Xiong W. Wong P. (2010). BACE1 deficiency causes altered neuronal activity and neurodegeneration. *J. Neurosci.* 30 8819–8829. 10.1523/JNEUROSCI.1334-10.2010 20592204 PMC2902368

[B47] Ikeda M. Takahashi N. Saito S. Aleksic B. Watanabe Y. Nunokawa A. (2008). Failure to replicate the association between NRG1 and schizophrenia using Japanese large sample. *Schizophr. Res.* 101 1–8. 10.1016/j.schres.2008.01.010 18282690

[B48] Ingason A. Soeby K. Timm S. Wang A. G. Jakobsen K. D. Fink-Jensen A. (2006). No significant association of the 5’ end of neuregulin 1 and schizophrenia in a large Danish sample. *Schizophr. Res.* 83 1–5. 10.1016/j.schres.2005.12.850 16483744

[B49] Javitt D. C. Lindsley R. W. (2001). Effects of phencyclidine on prepulse inhibition of acoustic startle response in the macaque. *Psychopharmacology* 156 165–168. 10.1007/s002130100758 11549218

[B50] Jiang J. Lin M. Xu Y. Shao J. Li X. Zhang H. (2016). Circulating neuregulin 4 levels are inversely associated with subclinical cardiovascular disease in obese adults. *Sci. Rep.* 6:36710 10.1038/srep36710 PMC5098181 27819316

[B51] Jiang L. Emmetsberger J. Talmage D. A. Role L. W. (2013). Type III neuregulin 1 is required for multiple forms of excitatory synaptic plasticity of mouse cortico-amygdala circuits. *J. Neurosci.* 33 9655–9666. 10.1523/JNEUROSCI.2888-12.2013 23739962 PMC3865493

[B52] Kamikubo Y. Takasugi N. Niisato K. Hashimoto Y. Sakurai T. (2017). Consecutive analysis of BACE1 function on developing and developed neuronal cells. *J. Alzheimers Dis.* 56 641–653. 10.3233/JAD-160806 28035928

[B53] Kao W. T. Wang Y. Kleinman J. E. Lipska B. K. Hyde T. M. Weinberger D. R. (2010). Common genetic variation in Neuregulin 3 (NRG3) influences risk for schizophrenia and impacts NRG3 expression in human brain. *Proc. Natl. Acad. Sci. U.S.A.* 107 15619–15624. 10.1073/pnas.1005410107 20713722 PMC2932571

[B54] Kennedy M. E. Stamford A. W. Chen X. Cox K. Cumming J. N. Dockendorf M. F. (2016). The BACE1 inhibitor verubecestat (MK-8931) reduces CNS beta-amyloid in animal models and in Alzheimer’s disease patients. *Sci. Transl. Med.* 8 363ra150 10.1126/scitranslmed.aad9704 27807285

[B55] Keri S. Beniczky S. Kelemen O. (2010). Suppression of the P50 evoked response and neuregulin 1-induced AKT phosphorylation in first-episode schizophrenia. *Am. J. Psychiatry* 167 444–450. 10.1176/appi.ajp.2009.09050723 20048019

[B56] Kim J. H. Park B. L. Pasaje C. F. Bae J. S. Park C. S. Cha B. (2012). Lack of associations of neuregulin 1 variations with schizophrenia and smooth pursuit eye movement abnormality in a Korean population. *J. Mol. Neurosci.* 46 476–482. 10.1007/s12031-011-9619-y 21858616

[B57] Kim J. W. Lee Y. S. Cho E. Y. Jang Y. L. Park D. Y. Choi K. S. (2006). Linkage and association of schizophrenia with genetic variations in the locus of neuregulin 1 in Korean population. *Am. J. Med. Genet. B Neuropsychiatr. Genet.* 141B 281–286. 10.1002/ajmg.b.30209 16526041

[B58] Knickmeyer R. C. Wang J. Zhu H. Geng X. Woolson S. Hamer R. M. (2014). Common variants in psychiatric risk genes predict brain structure at birth. *Cereb. Cortex* 24 1230–1246. 10.1093/cercor/bhs401 23283688 PMC3977618

[B59] Komossa K. Rummel-Kluge C. Schwarz S. Schmid F. Hunger H. Kissling W. (2011). Risperidone versus other atypical antipsychotics for schizophrenia. *Cochrane Database Syst. Rev.* CD006626 10.1002/14651858.CD006626.pub2 PMC4167865 21249678

[B60] Krivosheya D. Tapia L. Levinson J. N. Huang K. Kang Y. Hines R. (2008). ErbB4-neuregulin signaling modulates synapse development and dendritic arborization through distinct mechanisms. *J. Biol. Chem.* 283 32944–32956. 10.1074/jbc.M800073200 18819924 PMC5395083

[B61] Kubicki M. Westin C. F. Nestor P. G. Wible C. G. Frumin M. Maier S. E. (2003). Cingulate fasciculus integrity disruption in schizophrenia: a magnetic resonance diffusion tensor imaging study. *Biol. Psychiatry* 54 1171–1180. 10.1016/S0006-3223(03)00419-0 14643084 PMC2806222

[B62] Kuhn P. H. Koroniak K. Hogl S. Colombo A. Zeitschel U. Willem M. (2012). Secretome protein enrichment identifies physiological BACE1 protease substrates in neurons. *EMBO J.* 31 3157–3168. 10.1038/emboj.2012.173 22728825 PMC3400020

[B63] Kukshal P. Bhatia T. Bhagwat A. M. Gur R. E. Gur R. C. Deshpande S. N. (2013). Association study of neuregulin-1 gene polymorphisms in a North Indian schizophrenia sample. *Schizophr. Res.* 144 24–30. 10.1016/j.schres.2012.12.017 23360725 PMC4040109

[B64] Kumari V. Das M. Zachariah E. Ettinger U. Sharma T. (2005). Reduced prepulse inhibition in unaffected siblings of schizophrenia patients. *Psychophysiology* 42 588–594. 10.1111/j.0048-5772.2005.00346.x 16176381

[B65] Kwon O. B. Longart M. Vullhorst D. Hoffman D. A. Buonanno A. (2005). Neuregulin-1 reverses long-term potentiation at CA1 hippocampal synapses. *J. Neurosci.* 25 9378–9383. 10.1523/JNEUROSCI.2100-05.2005 16221846 PMC6725708

[B66] Lencer R. Trillenberg-Krecker K. Schwinger E. Arolt V. (2003). Schizophrenia spectrum disorders and eye tracking dysfunction in singleton and multiplex schizophrenia families. *Schizophr. Res.* 60 33–45. 10.1016/S0920-9964(02)00165-2 12505136

[B67] Li T. Stefansson H. Gudfinnsson E. Cai G. Liu X. Murray R. M. (2004). Identification of a novel neuregulin 1 at-risk haplotype in Han schizophrenia Chinese patients, but no association with the Icelandic/Scottish risk haplotype. *Mol. Psychiatry* 9 698–704. 10.1038/sj.mp.4001485 15007393

[B68] Lim K. O. Tew W. Kushner M. Chow K. Matsumoto B. DeLisi L. E. (1996). Cortical gray matter volume deficit in patients with first-episode schizophrenia. *Am. J. Psychiatry* 153 1548–1553. 10.1176/ajp.153.12.1548 8942450

[B69] Liu Y. Ford B. Mann M. A. Fischbach G. D. (2001). Neuregulins increase alpha7 nicotinic acetylcholine receptors and enhance excitatory synaptic transmission in GABAergic interneurons of the hippocampus. *J. Neurosci.* 21 5660–5669. 11466437 10.1523/JNEUROSCI.21-15-05660.2001 PMC6762647

[B70] Luo X. He W. Hu X. Yan R. (2014). Reversible overexpression of bace1-cleaved neuregulin-1 N-terminal fragment induces schizophrenia-like phenotypes in mice. *Biol. Psychiatry* 76 120–127. 10.1016/j.biopsych.2013.09.026 24210810 PMC3976896

[B71] Luo X. Prior M. He W. Hu X. Tang X. Shen W. (2011). Cleavage of neuregulin-1 by BACE1 or ADAM10 protein produces differential effects on myelination. *J. Biol. Chem.* 286 23967–23974. 10.1074/jbc.M111.251538 21576249 PMC3129178

[B72] Maher A. R. Theodore G. (2012). Summary of the comparative effectiveness review on off-label use of atypical antipsychotics. *J. Manag. Care Pharm.* 18(5 Suppl. B) S1–S20. 10.18553/jmcp.2012.18.s5-b.1 PMC10438344 22784311

[B73] Marballi K. Cruz D. Thompson P. Walss-Bass C. (2012). Differential neuregulin 1 cleavage in the prefrontal cortex and hippocampus in schizophrenia and bipolar disorder: preliminary findings. *PLOS ONE* 7:e36431 10.1371/journal.pone.0036431 PMC3349664 22590542

[B74] McIntosh A. M. Moorhead T. W. Job D. Lymer G. K. Munoz Maniega S. McKirdy J. (2008). The effects of a neuregulin 1 variant on white matter density and integrity. *Mol. Psychiatry* 13 1054–1059. 10.1038/sj.mp.4002103 17925794

[B75] Mei L. Xiong W. C. (2008). Neuregulin 1 in neural development, synaptic plasticity and schizophrenia. *Nat. Rev. Neurosci.* 9 437–452. 10.1038/nrn2392 18478032 PMC2682371

[B76] Meyhofer I. Steffens M. Kasparbauer A. Grant P. Weber B. Ettinger U. (2015). Neural mechanisms of smooth pursuit eye movements in schizotypy. *Hum. Brain Mapp.* 36 340–353. 10.1002/hbm.22632 25197013 PMC6869106

[B77] Michailov G. V. Sereda M. W. Brinkmann B. G. Fischer T. M. Haug B. Birchmeier C. (2004). Axonal neuregulin-1 regulates myelin sheath thickness. *Science* 304 700–703. 10.1126/science.1095862 15044753

[B78] Miyamoto Y. Torii T. Tanoue A. Kawahara K. Arai M. Tsumura H. (2017). Neuregulin-1 type III knockout mice exhibit delayed migration of Schwann cell precursors. *Biochem. Biophys. Res. Commun.* 486 506–513. 10.1016/j.bbrc.2017.03.074 28322798

[B79] Nason M. W. Jr. Adhikari A. Bozinoski M. Gordon J. A. Role L. W. (2011). Disrupted activity in the hippocampal-accumbens circuit of type III neuregulin 1 mutant mice. *Neuropsychopharmacology* 36 488–496. 10.1038/npp.2010.180 20927045 PMC3005939

[B80] Nave K. A. Ehrenreich H. (2014). Myelination and oligodendrocyte functions in psychiatric diseases. *JAMA Psychiatry* 71 582–584. 10.1001/jamapsychiatry.2014.189 24671770

[B81] Nave K. A. Salzer J. L. (2006). Axonal regulation of myelination by neuregulin 1. *Curr. Opin. Neurobiol.* 16 492–500. 10.1016/j.conb.2006.08.008 16962312

[B82] Naz M. Riaz M. Saleem M. (2011). Potential role of Neuregulin 1 and TNF-alpha (-308) polymorphism in schizophrenia patients visiting hospitals in Lahore, Pakistan. *Mol. Biol. Rep.* 38 4709–4714. 10.1007/s11033-010-0606-0 21127983

[B83] Newell K. A. Karl T. Huang X. F. (2013). A neuregulin 1 transmembrane domain mutation causes imbalanced glutamatergic and dopaminergic receptor expression in mice. *Neuroscience* 248 670–680. 10.1016/j.neuroscience.2013.06.037 23811072

[B84] Oh H. J. Nam B. Y. Lee M. J. Kim C. H. Koo H. M. Doh F. M. (2015). Decreased circulating klotho levels in patients undergoing dialysis and relationship to oxidative stress and inflammation. *Perit. Dial. Int.* 35 43–51. 10.3747/pdi.2013.00150 24497597 PMC4335927

[B85] Pan B. Huang X. F. Deng C. (2011). Antipsychotic treatment and neuregulin 1-ErbB4 signalling in schizophrenia. *Prog. Neuropsychopharmacol. Biol. Psychiatry* 35 924–930. 10.1016/j.pnpbp.2011.04.002 21513767

[B86] Papaleo F. Yang F. Paterson C. Palumbo S. Carr G. V. Wang Y. (2016). Behavioral, neurophysiological, and synaptic impairment in a transgenic neuregulin1 (NRG1-IV) murine schizophrenia model. *J. Neurosci.* 36 4859–4875. 10.1523/JNEUROSCI.4632-15.2016 27122041 PMC4846677

[B87] Papiol S. Begemann M. Rosenberger A. Friedrichs H. Ribbe K. Grube S. (2011). A phenotype-based genetic association study reveals the contribution of neuregulin1 gene variants to age of onset and positive symptom severity in schizophrenia. *Am. J. Med. Genet. B Neuropsychiatr. Genet.* 156B 340–345. 10.1002/ajmg.b.31168 21234898

[B88] Park S. K. Solomon D. Vartanian T. (2001). Growth factor control of CNS myelination. *Dev. Neurosci.* 23 327–337. 10.1159/000048716 11756748

[B89] Pasaje C. F. Bae J. S. Park B. L. Cheong H. S. Kim J. H. Park T. J. (2011). Neuregulin 3 does not confer risk for schizophrenia and smooth pursuit eye movement abnormality in a Korean population. *Genes Brain Behav.* 10 828–833. 10.1111/j.1601-183X.2011.00722.x 21762460

[B90] Polich J. Kok A. (1995). Cognitive and biological determinants of P300: an integrative review. *Biol. Psychol.* 41 103–146. 10.1016/0301-0511(95)05130-9 8534788

[B91] Rethelyi J. M. Bakker S. C. Polgar P. Czobor P. Strengman E. Pasztor P. I. (2010). Association study of NRG1, DTNBP1, RGS4, G72/G30, and PIP5K2A with schizophrenia and symptom severity in a Hungarian sample. *Am. J. Med. Genet. B Neuropsychiatr. Genet.* 153B 792–801. 10.1002/ajmg.b.31049 19937977

[B92] Rieff H. I. Raetzman L. T. Sapp D. W. Yeh H. H. Siegel R. E. Corfas G. (1999). Neuregulin induces GABA(A) receptor subunit expression and neurite outgrowth in cerebellar granule cells. *J. Neurosci.* 19 10757–10766. 10594059 10.1523/JNEUROSCI.19-24-10757.1999 PMC6784934

[B93] Rimer M. Barrett D. W. Maldonado M. A. Vock V. M. Gonzalez-Lima F. (2005). Neuregulin-1 immunoglobulin-like domain mutant mice: clozapine sensitivity and impaired latent inhibition. *Neuroreport* 16 271–275. 10.1097/00001756-200502280-00014 15706234

[B94] Rio C. Rieff H. I. Qi P. Khurana T. S. Corfas G. (1997). Neuregulin and erbB receptors play a critical role in neuronal migration. *Neuron* 19 39–50. 10.1016/S0896-6273(00)80346-3 9247262

[B95] Roussos P. Giakoumaki S. G. Adamaki E. Bitsios P. (2011). The influence of schizophrenia-related neuregulin-1 polymorphisms on sensorimotor gating in healthy males. *Biol. Psychiatry* 69 479–486. 10.1016/j.biopsych.2010.09.009 21035784

[B96] Savonenko A. V. Melnikova T. Laird F. M. Stewart K. A. Price D. L. Wong P. C. (2008). Alteration of BACE1-dependent NRG1/ErbB4 signaling and schizophrenia-like phenotypes in BACE1-null mice. *Proc. Natl. Acad. Sci. U.S.A.* 105 5585–5590. 10.1073/pnas.0710373105 18385378 PMC2291091

[B97] Schizophrenia Working Group of the Psychiatric Genomics Consortium (2014). Biological insights from 108 schizophrenia-associated genetic loci. *Nature* 511 421–427. 10.1038/nature13595 25056061 PMC4112379

[B98] Schmechtig A. Vassos E. Kumari V. Hutton S. B. Collier D. A. Morris R. G. (2010). Association of Neuregulin 1 rs3924999 genotype with antisaccades and smooth pursuit eye movements. *Genes Brain Behav.* 9 621–627. 10.1111/j.1601-183X.2010.00594.x 20497232

[B99] Schmid R. S. McGrath B. Berechid B. E. Boyles B. Marchionni M. Sestan N. (2003). Neuregulin 1-erbB2 signaling is required for the establishment of radial glia and their transformation into astrocytes in cerebral cortex. *Proc. Natl. Acad. Sci. U.S.A.* 100 4251–4256. 10.1073/pnas.0630496100 12649319 PMC153079

[B100] Schultz S. H. North S. W. Shields C. G. (2007). Schizophrenia: a review. *Am. Fam. Physician* 75 1821–1829. 17619525

[B101] Seshadri S. Kamiya A. Yokota Y. Prikulis I. Kano S. Hayashi-Takagi A. (2010). Disrupted-in-Schizophrenia-1 expression is regulated by beta-site amyloid precursor protein cleaving enzyme-1-neuregulin cascade. *Proc. Natl. Acad. Sci. U.S.A.* 107 5622–5627. 10.1073/pnas.0909284107 20212127 PMC2851766

[B102] Shaikh M. Hall M. H. Schulze K. Dutt A. Walshe M. Williams I. (2011). Do COMT, BDNF and NRG1 polymorphisms influence P50 sensory gating in psychosis? *Psychol. Med.* 41 263–276. 10.1017/S003329170999239X 20102668

[B103] Shibuya M. Komi E. Wang R. Kato T. Watanabe Y. Sakai M. (2010). Measurement and comparison of serum neuregulin 1 immunoreactivity in control subjects and patients with schizophrenia: an influence of its genetic polymorphism. *J. Neural Transm.* 117 887–895. 10.1007/s00702-010-0418-3 20526724

[B104] Shiota S. Tochigi M. Shimada H. Ohashi J. Kasai K. Kato N. (2008). Association and interaction analyses of NRG1 and ERBB4 genes with schizophrenia in a Japanese population. *J. Hum. Genet.* 53 929–935. 10.1007/s10038-008-0332-9 18704261

[B105] Smyrnis N. Kattoulas E. Stefanis N. C. Avramopoulos D. Stefanis C. N. Evdokimidis I. (2011). Schizophrenia-related neuregulin-1 single-nucleotide polymorphisms lead to deficient smooth eye pursuit in a large sample of young men. *Schizophr. Bull.* 37 822–831. 10.1093/schbul/sbp150 19965935 PMC3122292

[B106] Squassina A. Piccardi P. Del Zompo M. Rossi A. Vita A. Pini S. (2010). NRG1 and BDNF genes in schizophrenia: an association study in an Italian case-control sample. *Psychiatry Res.* 176 82–84. 10.1016/j.psychres.2009.03.017 20061032

[B107] Stassart R. M. Fledrich R. Velanac V. Brinkmann B. G. Schwab M. H. Meijer D. (2013). A role for Schwann cell-derived neuregulin-1 in remyelination. *Nat. Neurosci.* 16 48–54. 10.1038/nn.3281 23222914

[B108] Stedehouder J. Kushner S. A. (2017). Myelination of parvalbumin interneurons: a parsimonious locus of pathophysiological convergence in schizophrenia. *Mol. Psychiatry* 22 4–12. 10.1038/mp.2016.147 27646261 PMC5414080

[B109] Stefansson H. Sarginson J. Kong A. Yates P. Steinthorsdottir V. Gudfinnsson E. (2003). Association of neuregulin 1 with schizophrenia confirmed in a Scottish population. *Am. J. Hum. Genet.* 72 83–87. 10.1086/345442 12478479 PMC420015

[B110] Stefansson H. Sigurdsson E. Steinthorsdottir V. Bjornsdottir S. Sigmundsson T. Ghosh S. (2002). Neuregulin 1 and susceptibility to schizophrenia. *Am. J. Hum. Genet.* 71 877–892. 10.1086/342734 12145742 PMC378543

[B111] Storozheva Z. I. Kirenskaya A. V. Novototsky-Vlasov V. Y. Telesheva K. Y. Pletnikov M. (2016). Startle modification and P50 gating in schizophrenia patients and controls: Russian population. *Span. J. Psychol.* 19 E8 10.1017/sjp.2016.1 26936103

[B112] Sun Z. Wang F. Cui L. Breeze J. Du X. Wang X. (2003). Abnormal anterior cingulum in patients with schizophrenia: a diffusion tensor imaging study. *Neuroreport* 14 1833–1836. 10.1097/01.wnr.0000094529.75712.48 14534430

[B113] Thirunavukkarasu P. Vijayakumari A. A. John J. P. Halahalli H. N. Paul P. Sen S. (2014). An exploratory association study of the influence of dysbindin and neuregulin polymorphisms on brain morphometry in patients with schizophrenia and healthy subjects from South India. *Asian J. Psychiatry* 10 62–68. 10.1016/j.ajp.2014.04.002 25042954

[B114] Thomson P. A. Christoforou A. Morris S. W. Adie E. Pickard B. S. Porteous D. J. (2007). Association of Neuregulin 1 with schizophrenia and bipolar disorder in a second cohort from the Scottish population. *Mol. Psychiatry* 12 94–104. 10.1038/sj.mp.4001889 16940976

[B115] Timmers M. Barao S. Van Broeck B. Tesseur I. Slemmon J. De Waepenaert K. (2017). BACE1 dynamics upon inhibition with a BACE inhibitor and correlation to downstream Alzheimer’s disease markers in elderly healthy participants. *J. Alzheimers Dis.* 56 1437–1449. 10.3233/JAD-160829 28157093 PMC5325057

[B116] Tosato S. Bellani M. Bonetto C. Ruggeri M. Perlini C. Lasalvia A. (2012). Is neuregulin 1 involved in determining cerebral volumes in schizophrenia? Preliminary results showing a decrease in superior temporal gyrus volume. *Neuropsychobiology* 65 119–125. 10.1159/000330584 22378022

[B117] Turetsky B. I. Calkins M. E. Light G. A. Olincy A. Radant A. D. Swerdlow N. R. (2007). Neurophysiological endophenotypes of schizophrenia: the viability of selected candidate measures. *Schizophr. Bull.* 33 69–94. 10.1093/schbul/sbl060 17135482 PMC2632291

[B118] Turunen J. A. Peltonen J. O. Pietilainen O. P. Hennah W. Loukola A. Paunio T. (2007). The role of DTNBP1, NRG1, and AKT1 in the genetics of schizophrenia in Finland. *Schizophr. Res.* 91 27–36. 10.1016/j.schres.2006.11.028 17300918

[B119] van Os J. Kapur S. (2009). Schizophrenia. *Lancet* 374 635–645. 10.1016/S0140-6736(09)60995-8 19700006

[B120] Voineskos A. N. Felsky D. Kovacevic N. Tiwari A. K. Zai C. Chakravarty M. M. (2013). Oligodendrocyte genes, white matter tract integrity, and cognition in schizophrenia. *Cereb. Cortex* 23 2044–2057. 10.1093/cercor/bhs188 22772651 PMC3729194

[B121] Vullhorst D. Ahmad T. Karavanova I. Keating C. Buonanno A. (2017). Structural similarities between neuregulin 1-3 isoforms determine their subcellular distribution and signaling mode in central neurons. *J. Neurosci.* 37 5232–5249. 10.1523/JNEUROSCI.2630-16.2017 28432142 PMC5456106

[B122] Wan L. Friedman B. H. Boutros N. N. Crawford H. J. (2008). P50 sensory gating and attentional performance. *Int. J. Psychophysiol.* 67 91–100. 10.1016/j.ijpsycho.2007.10.008 18036692 PMC2292346

[B123] Wan L. Thomas Z. Pisipati S. Jarvis S. P. Boutros N. N. (2017). Inhibitory deficits in prepulse inhibition, sensory gating, and antisaccade eye movement in schizotypy. *Int. J. Psychophysiol.* 114 47–54. 10.1016/j.ijpsycho.2017.02.003 28189549

[B124] Wang F. Jiang T. Sun Z. Teng S. L. Luo X. Zhu Z. (2009). Neuregulin 1 genetic variation and anterior cingulum integrity in patients with schizophrenia and healthy controls. *J. Psychiatry Neurosci.* 34 181–186. 19448847 PMC2674970

[B125] Wang F. Sun Z. Cui L. Du X. Wang X. Zhang H. (2004). Anterior cingulum abnormalities in male patients with schizophrenia determined through diffusion tensor imaging. *Am. J. Psychiatry* 161 573–575. 10.1176/appi.ajp.161.3.573 14992988

[B126] Wang G. X. Zhao X. Y. Meng Z. X. Kern M. Dietrich A. Chen Z. (2014). The brown fat-enriched secreted factor Nrg4 preserves metabolic homeostasis through attenuation of hepatic lipogenesis. *Nat. Med.* 20 1436–1443. 10.1038/nm.3713 25401691 PMC4257907

[B127] Wang H. Li R. Shen Y. (2013). beta-Secretase: its biology as a therapeutic target in diseases. *Trends Pharmacol. Sci.* 34 215–225. 10.1016/j.tips.2013.01.008 23452816 PMC3691103

[B128] Wang X. D. Su Y. A. Guo C. M. Yang Y. Si T. M. (2008). Chronic antipsychotic drug administration alters the expression of neuregulin 1beta, ErbB2, ErbB3, and ErbB4 in the rat prefrontal cortex and hippocampus. *Int. J. Neuropsychopharmacol.* 11 553–561. 10.1017/S1461145707008371 18184445

[B129] Wang Y. C. Chen J. Y. Chen M. L. Chen C. H. Lai I. C. Chen T. T. (2008). Neuregulin 3 genetic variations and susceptibility to schizophrenia in a Chinese population. *Biol. Psychiatry* 64 1093–1096. 10.1016/j.biopsych.2008.07.012 18708184

[B130] Wen D. Suggs S. V. Karunagaran D. Liu N. Cupples R. L. Luo Y. (1994). Structural and functional aspects of the multiplicity of Neu differentiation factors. *Mol. Cell. Biol.* 14 1909–1919. 10.1128/MCB.14.3.1909 7509448 PMC358549

[B131] Willem M. Garratt A. N. Novak B. Citron M. Kaufmann S. Rittger A. (2006). Control of peripheral nerve myelination by the beta-secretase BACE1. *Science* 314 664–666. 10.1126/science.1132341 16990514

[B132] Winterer G. Konrad A. Vucurevic G. Musso F. Stoeter P. Dahmen N. (2008). Association of 5’ end neuregulin-1 (NRG1) gene variation with subcortical medial frontal microstructure in humans. *Neuroimage* 40 712–718. 10.1016/j.neuroimage.2007.12.041 18255317

[B133] Yan L. Shamir A. Skirzewski M. Leiva-Salcedo E. Kwon O. B. Karavanova I. (2017). Neuregulin-2 ablation results in dopamine dysregulation and severe behavioral phenotypes relevant to psychiatric disorders. *Mol. Psychiatry* 10.1038/mp.2017.22 [Epub ahead of print]. PMC5608621 28322273

[B134] Yang J. Z. Si T. M. Ruan Y. Ling Y. S. Han Y. H. Wang X. L. (2003). Association study of neuregulin 1 gene with schizophrenia. *Mol. Psychiatry* 8 706–709. 10.1038/sj.mp.4001377 12874607

[B135] Yarden Y. Sliwkowski M. X. (2001). Untangling the ErbB signalling network. *Nat. Rev. Mol. Cell Biol.* 2 127–137. 10.1038/35052073 11252954

[B136] Yoosefee S. Shahsavand Ananloo E. Joghataei M. T. Karimipour M. Hadjighassem M. Mohaghghegh H. (2016). Association between neuregulin-1 gene variant (rs2439272) and schizophrenia and its negative symptoms in an Iranian population. *Iran. J. Psychiatry* 11 147–153. 27928246 PMC5139949

[B137] Zhang D. Sliwkowski M. X. Mark M. Frantz G. Akita R. Sun Y. (1997). Neuregulin-3 (NRG3): a novel neural tissue-enriched protein that binds and activates ErbB4. *Proc. Natl. Acad. Sci. U.S.A.* 94 9562–9567. 10.1073/pnas.94.18.9562 9275162 PMC23218

[B138] Zhang H. X. Zhao J. P. Lv L. X. Li W. Q. Xu L. Ouyang X. (2008). Explorative study on the expression of neuregulin-1 gene in peripheral blood of schizophrenia. *Neurosci. Lett.* 438 1–5. 10.1016/j.neulet.2007.09.051 18455303

[B139] Zhou L. Barao S. Laga M. Bockstael K. Borgers M. Gijsen H. (2012). The neural cell adhesion molecules L1 and CHL1 are cleaved by BACE1 protease in vivo. *J. Biol. Chem.* 287 25927–25940. 10.1074/jbc.M112.377465 22692213 PMC3406677

